# From the heart of the animal feed industry: a Southeast Asian perspective on insects for feed in Asia

**DOI:** 10.1093/af/vfad036

**Published:** 2023-08-14

**Authors:** Anne Deguerry, Nathan Preteseille, Attawit Kovitvadhi, David John Allan, Sonevilay Nampanya, Scott Newman

**Keywords:** Black Soldier Fly, insects as feed, market, regulation, Southeast Asia

**Implications**

- Insects as feed in Southeast Asia is a nascent industry with very diverse features.
- Academic research in Thailand in the insect sector could be a regional driver to fill the knowledge gap.
- Asia is suitable for various models of development. A clear regulatory landscape, incentives from the market, support from the investment funds, and R&D from the academia and the industry will be able to support, promote, and accelerate the development of an inclusive and sustainable insect as feed industry in the region.

During 2022, three of the authors (Allan, Deguerry, and Preteseille) have been in charge of the management of the project: *Understanding the use of Insect-based protein source for animal and aquaculture feeds in Asia and the Pacific region*, a collaboration between FAO (Newman and Nampanya), Kasetsart University (KU), and AFFIA. This study, prepared for and with FAO support, aims at giving an overall picture of insects as feed in Asia. The regional survey mentioned refers to a component of the overall project, it gathered answers from 41 stakeholders. The results and conclusions of the study have served as a part of the reference for the development of this article, which aims to highlight the growing role of insects in the Asian feed industry.

## Introduction: Insects as Feed in Asia—Consideration and Limitations

Asia-Pacific regional animal feed production has had the fastest global growth in 2022. While, in most segments, China and India are the driving countries, noticeable leadership was taken by other countries. For instance, Indonesia accounted for 10% of Asia-Pacific’s growth in the aquaculture feed sector in 2022 (Alltech, 2022).

It is important to clarify the scope of the regional definition: Asia, Asia-Pacific, and Southeast Asia represent very different geographies with a major impact on market evaluation. As a basis for this article, we have considered countries mainly in Southeast Asia as well as India. The countries are where Asian Food and Feed Insect Association (AFFIA) has members and also the authors have experience. Current AFFIA membership does not have enough representation to allow a comprehensive view of the Asia-Pacific insect as feed industry. Major sector developments underway in countries such as the Republic of Korea, Japan, or Australia have not been included, and information on China is scant and has not been covered by the survey work referred to above.

Whichever part of the world we observe, disparities will amplify as we zoom in to the regional level and Asia is no exception. While we reduce the study scope to AFFIA’s geographical membership footprint, the historical, geographical, economic, regulatory, academic, and industrial factors draw a mosaic of diversity, where some caveats should be noted.

We recognize that the lack of information for important countries hinders the possibility of extrapolating interpretation at the regional level (e.g., the Republic of Korea and Japan). China presents particular difficulties as the largest feed-producing country with the most important growth in animal feed production since 2020 (Alltech, 2022) yet so little reliable data on insects for feed are available to date.

Applied academic studies and information availability are key to encouraging the use of insect-based products in compound feed, yet competitiveness issues in the new industry can retard information sharing.

## A Historical Leadership

Entomophagy exists throughout the world and Southeast Asia has had a long and strong yet heterogeneous tradition of insect consumption, and insects are still eaten today in many areas. Thailand is remarkable for its large-scale consumption of a wide variety of insects and can be considered the worldwide cradle of insect farming, having developed an insect farming industry since 1997 (Halloran et al., 2016), with over 20,000 insect farms reported (Hanboonsong et al., 2013) for production around 7,500 tons per year (op. cit. data from 1996 to 2011). It was the first country in the world to release guidelines for cricket farming in 2017 (Cricket GAP, cf. infra).

In the field of insect as feed, it is important to acknowledge the prominence of the pioneer Black Soldier Fly (BSF) projects in the region, such as those developed in Indonesia by the French Institut de Recherche pour le Developpement (Caruso et al., 2014) and by the Swiss Federal Institute in Aquatic Research EAWAG (practical know-how on BSF processing) EAWAG (2018). Those organizations have published open-access information and technical manuals, which has enabled newcomers to start BSF farming with a solid foundation.

**Figure 1.**
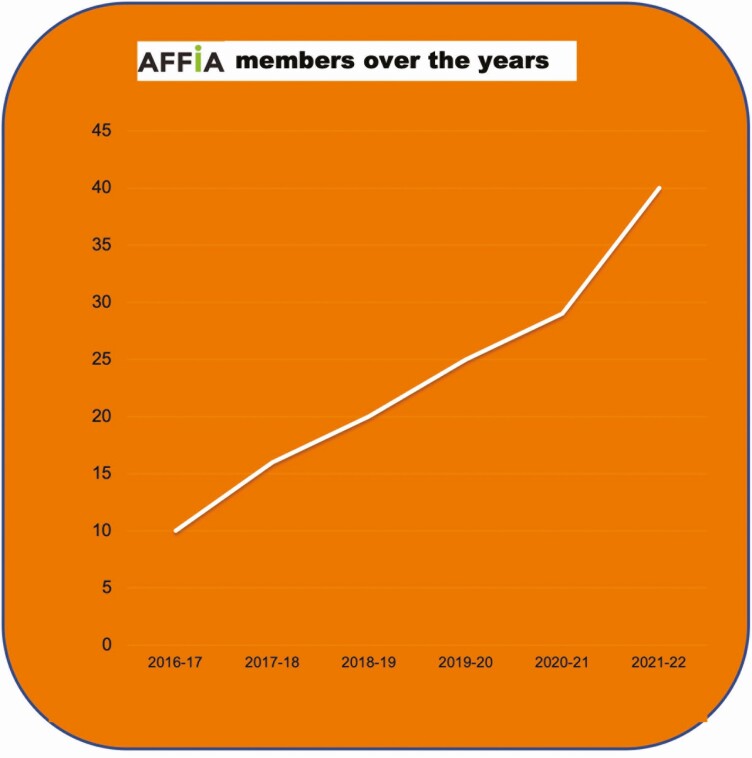
Members of AFFIA over the years. Source: AFFIA.

By August 2016, the idea of forming a collaborative platform to support the development of the insect industry in the region had germinated. Driven by Thai actors, including Kasetsart University, the regional stakeholders met in Bangkok to create the group that would give birth to the regional association AFFIA. The group was started with 10 members from the industry and research sectors, including some pioneering BSF projects in the region: EAWAG in Indonesia, ENTOBEL in Vietnam, and ENTOFOOD in Malaysia. The following year, AFFIA brought together 15 members from 8 Asian countries (Preteseille, 2018; cf. Figure 1).

The development of insect farming dedicated to the feed industry began much later than its food counterpart. Thus, the regional survey indicates that the majority of companies surveyed started to commercialize their production from 2020 onwards.

### Insects and the BSF

While some other species, such as fruit flies or silkworms, are produced and studied, the BSF is the predominant species farmed by nearly 90% of the surveyed stakeholders. BSF has remarkable zootechnical traits: short life cycle, high fecundity, wide range of acceptable diet, global presence, and high nutritional value combined with a noninvasive, nonpathogenic, and nonpest status brought the BSF into the spotlight of the insect as feed industry.

It is of key importance to realize the diversity of insect species, life cycles, and potential applications starting with nutrition (Oonincx and Finke, 2020). Holometabolous (Figure 4) vs. Hemimetabolous species already provide an important level of challenges to understand and possibly farm and further upscale production. The BSF has to date been the most studied and understood, yet the potential is close to infinite to catch the ecosystem services insects may provide in their diversity and complexity (Jongema, 2017).

## Regulatory Context

Asia includes an important diversity of development statutes and consequently regulatory development levels, which are also true in the context of the Association of Southeast Asian Nations (ASEAN). The AFFIA was originally created with the ambition to speak as one voice for the insect industry in Southeast Asia to regional regulatory bodies, as the image of its global counterparts in their respective regions, such as the Insect Protein Association of Australia (IPAA), the International Platform for Insects as Food and Feed (IPIFF) in Europe, or the North American Coalition for Insect Agriculture (NACIA).

It rapidly became clear that national frameworks were to lead possible regional adoptions in line with specific insect industry experiences and market needs. For example, in Thailand, where the farming of crickets already showed several decades of unique experience, Good Agricultural Practices (GAP) for several cricket species were established (2017), driven by the export market potential. Yet this has not been adopted by other ASEAN countries to date.

A few years later, Singapore developed dedicated regulations for its insect industry to produce and market BSF and other authorized species from farm to table, with a recent public consultation towards continuous improvement of the rules (December 2022).

While this heterogenicity presents enormous opportunities, it also triggers multiple challenges for stakeholders to produce and trade insect-based products for food and feed. It is difficult not to correlate this with investment in the Asian insect industry. In most cases, the status or even the presence of the main species seen in the industry is yet to be recognized by regulatory frameworks, creating a relatively high risk for companies deciding to invest in production premises in Southeast Asia.

The lack of necessary requirements also puts good practitioners at risk of facing the burden of building a safe and sustainable image for the industry without safeguards in place to protect against opportunistic approaches.

When looking at the regulatory framework, one could split the approach into three parts for a given country: 1) production, 2) placement on the market (import and domestic), and 3) export. In brief, we can recap for the region as follows:

(1) Few insect-dedicated rules are evident except for the cases of Singapore and Thailand (voluntary GAP). Despite clear regulations in place regarding pest management in most countries, authorized species for farming are yet to be defined as well as feedstocks.
(2) Rules applying to the placement on the market of corresponding target applications often constitute current practices, following existing rules on food and feed products or ingredients.
(3) Export requirements are mainly driven by the targeted export country and are increasingly adopted by producers focusing on this market. Such is the case for the European Union, which is considered important for insect manufacturers worldwide.

Key regional advantages for the Asian insect industry are: (1) the historical experience of Southeast Asia with the production, use of insects, and their applications, (2) the tropical environment recognized as favorable for production, and (3) the strategic position at the core of the food and feed industry’s growth. For the maturation of a safe, inclusive, and sustainable industry in Southeast Asia, it will be necessary to develop the right regulatory landscape and policy support to enhance the establishment and development of insect production. This could be enabled through the following noncomprehensive list of initial measures:

- The clear identification of responsible regulatory bodies for insect industry value chains.
- The clear regulatory recognition of dedicated species authorized for food and feed production.
- The identification of authorized feedstocks based on regional resources and science-based approaches.
- The development of regional collaboration to establish mutually recognized requirements and regionally build a global insect industry hotspot.
- The development of incentives for the market to increasingly consider insects as relevant ingredients in its supply chain.

The Asian insect industry has already proven its potential—regulation should be part of the key enablers to assist.

## Research and academic context: the example of Thailand

**Figure 2.**
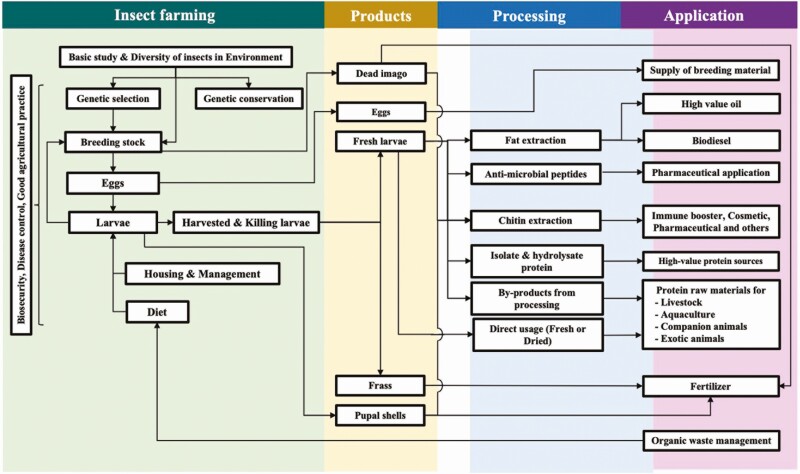
Research aspects of BSF about farming, products, processing, and their applications in Thailand.

The body of insect as feed studies is growing fast and the number of publications about industrial insect species in Thailand increased from 21 in 2015–2019 to 49 in 2020–2021, indicating a growing interest among researchers. Additionally, research funding in Thailand supports the study and investment in various aspects of the insect industry. Due to Thailand’s diverse biodiversity, insects such as crickets, mulberry silkworms, and sago palm weevils are farmed for human consumption, but are not cost-effective for use as feed. Therefore, BSF is considered a promising option for industrial-scale production. Figure 2 provides an overview of research on BSF in Thailand.

Eggs of BSF for breeding colonies are typically collected from the environment after baiting. To promote sustainability, genetic selection and conservation could be performed. Studies have been conducted on the genetic structure of BSF from Thailand and other countries to gather data for conservation and diversity (Kaya et al., 2021). Additionally, research is being done on the whole and functional genomics of BSF to identify key components, which may lead to novel efficiencies. The main source of knowledge on housing, management, and automatic systems for BSF comes from the industrial sector. As found in a study by Zhen et al. (2020), compared to freezing, carbon dioxide, or vacuum, the recommended method for killing BSF is blanching in boiling water for 40 s before drying. There remains a need for more research on the management of heat-, air-, and water-based pollutants during cultivation to minimize environmental impact.

The activities of amylase and protease are high in the larval stage, whereas cellulase activity is low (Intayung et al., 2021). Therefore, the degradation of fiber is done by exogenous microbes. Industry by-products such as coconut endosperm, soybean curd residue, blended sewage sludge, palm kernel spent pressings, and organic waste can be used as feed for BSF larvae to reduce costs (Pliantiangtam et al., 2021; Raksasat et al., 2021; Wong et al., 2021). The appropriate ratio of carbon to nitrogen (Pang et al., 2020) in diets must be formulated to obtain high quality and quantity. Moreover, it is important to control the specifications and characteristics of insect-based products in large-scale industrial production. Additionally, a growth model of BSF larvae has been developed to estimate their diet quality (Sripontan et al., 2020). The guidelines on biosecurity management, disease control, and good agricultural practices could be announced for producers in small- and large-scale production, following the lead of those previously developed for the cricket industry.

There are several applications for the products from BSF, such as high-value oil, biodiesel, antimicrobial peptide, chitin, and protein isolate and hydrolysate. Most insect ingredients are suitable for animal feed and have similar digestibility to soybean meal in broilers and quails based on in vitro digestibility (Kovitvadhi et al., 2021). BSF larvae can be fed to Thai climbing perch (Mapanao et al., 2021) and Nile tilapia (Tippayadara et al., 2021) without negative consequences by replacing the fish meal at 75% and 100%, respectively. Studies are ongoing to use BSF larvae as feed for poultry, swine, aquatic animals, dogs, cats, and exotic pets. The residual material (frass) after larval harvesting can be used as fertilizer and/or soil conditioner. Additionally, the pupal shells and dead adults can be used for other products, such as biomaterials (cf. infra).

Thailand’s researchers have been focusing on various aspects of BSF larvae and its application with the support of governmental grants and policies to enable Thailand’s potential as a regional leader in the insect as food and feed industry.

## Market Dynamics

When addressing the animal feed industry markets, key constraints such as volume, quality, and price prevail in the selection of ingredients. Insect products from BSF can be used for most farmed species and especially monogastric animals, as a protein source, as additives in feed formulation or even in direct feeding applications. Since statistics are available for compound feed, they provide a reference in terms of potential markets. In 2021, the global feed industry represented a total production of 1.2 billion tons (Alltech, 2022). China represents the world’s largest feed producer with 261,000,000 tons. The rest of Asia contributes nearly another 200,000,000 tons. Tentatively forecasting a potential market share for insect meal in Asia leads us to potentially hundreds of thousands of tons considering targeted market segments (pet food, aquafeed, livestock feed) and reported inclusion levels (feed 5% to 10% and pet food 10% to 30%).

From a production perspective and with 41 stakeholders, the 2022 AFFIA survey (to be published) highlighted a potential production of over 200,000 tons a year of fresh larvae by 2025. As these results come from forecasts with multiple challenges, further refinement of the data and careful consideration of their use are suggested. What is clearly highlighted is the large gap between nascent insect production and the huge, long-established animal feed market.

Large potential markets associated with a small volume of production usually represent a chicken and egg situation. Specialized markets have high purchasing power and will support growth and address demand gaps through improved perceptions of insects’ benefits (nutrition, functional performance, sustainability, and even “humanization” of pet and related diets). The survey highlighted the current focus of the regional producers on their respective domestic markets followed by international and regional ones.

In 2022, pet food tonnage reached almost 2,5 millions metric tons for the Asia-pacific region (Alltech, 2023). And pet food represented the leading market for internationally oriented producers, while aquaculture and livestock are the industries of focus when considering large-scale regional applications and markets. The region provides a unique opportunity to address demand gaps with a diversity of scales and formats of application in all market segments.

### Which trends for which markets?

**Figure 3.**
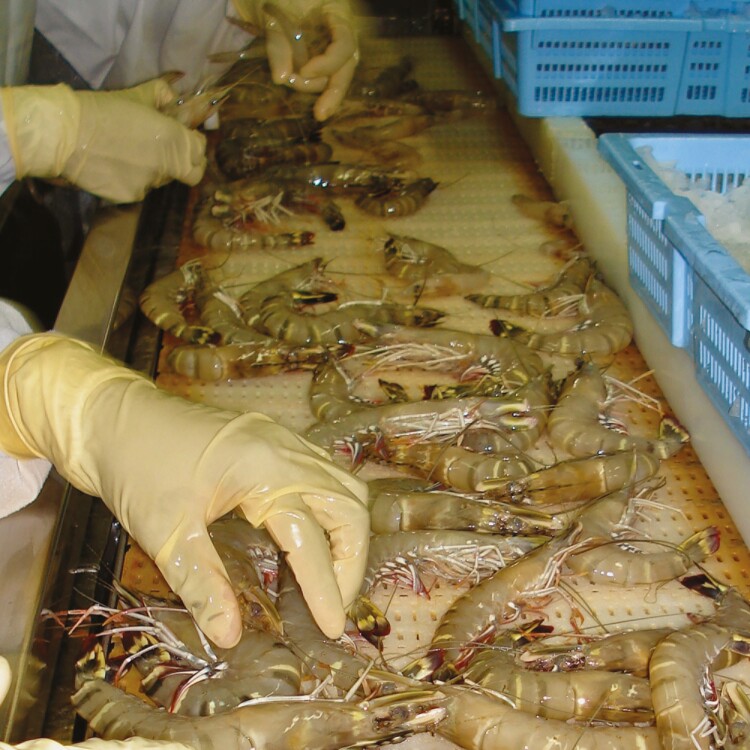
BSFL ingredients are triggering an increasing interest in shrimp feed. Here, farmed shrimp on the process line. © Entofood.

If we look at recent trials done by the industry in the region, either published, to be published, or presented at conferences, we notice a strong interest in the shrimp market as an important Asian aquaculture submarket (Figure 3). Four recent tests on whiteleg shrimp (Litopenaeus *vannamei*) were conducted:

- In 2021, Nutrition Technologies conducted an experiment to find the optimum inclusion rate of BSF to replace fish meal with a minimal impact in terms of costs. From 5% to 35% fish meal replacement, 25% appeared to have the best efficiency and cost ratio (Feed Navigator, 2021).
- In 2022, Entobel in Vietnam conducted a trial with Hue University (to be published) where BSF meal was tested on the growth effects and economics of shrimp farming. They state that partly replacing fish meal in shrimp feed with BSF meal leads to better growth, lower FCR, and higher final biomass, hence demonstrating the cost-effectiveness of including insect meal, even though the price of insect meal is higher than the fish meal it replaces.
- In Indonesia, Biocycle did a test with a low inclusion of BSF meal. According to their results, substitution levels of 2% and 5% of fish meal in the feed significantly improved growth and FCR (Info Akuakultur, September. 2022)
- Veolia also performed a trial with Kasetsart University with dietary inclusion of 2%, 5%, and 10% of BSF meal stand-alone or in combination with BSF oil (2% insect meal and 2% insect oil). The results have shown better growth of shrimp postlarvae and increased survival in juveniles, as well as improved FCR and gut health at levels of 5% and 10% insect meal inclusion and in combination with insect oil (Verstraete et al., 2023).

**Figure 4.**
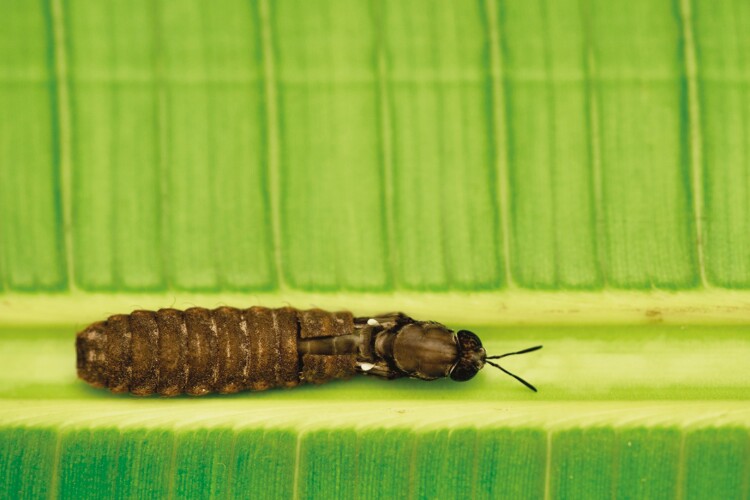
BSF is a holometabolous insect. Here, the adult fly is emerging from its pupae shell © Anne Deguerry.

In addition to the aquafeed market, the pet food market offers a niche positioning with a strong sustainability marketing argument (Siddiqui et al., 2023), which underlies the upward trend of the market. Applied research is carried out by local companies such as Protenga in Singapore (AFFIA Annual Conference 2022—Bangkok) about the palatability of the insect meal in dog’s nutrition. It is also important to consider that the pet food market is not limited to ordinary cats and dogs. In Asia, pet ownership is diverse, with strong markets existing for reptiles, amphibians, exotic mammals, ornamental fish, and songbirds (Devic, 2021).

In a completely different segment, innovative products are under development by Singaporean company, Insectta, which focuses on high-added value markets with biomaterial production such as chitosan and melanin (AFFIA Annual Conference 2022—Bangkok). Additionally, applied research is being conducted on organic semiconductors.

Beyond these examples and trends, the sector is ultimately shaped by internal and external factors. Authorization of feedstock practices, rapid learning curve progress, and increased adoption by the compound feed industry of insect-based ingredients are key to the development of the industry. Specialist predictions are that the long-term outlook will be focused around animal health, sustainability, and higher conversion efficiency (All About Feed, 2023). These three features are at the very heart of the relevance for considering the use of insect-based products and each represents key drivers for the Asian animal feed industry’s development.

### Models of development

Asia is suitable for various models of development and in particular to a two-speed model, where the large scale sits side by side with the small scale without interference between the two. The small producers are taking advantage of the virtuous circle of circularity at a very local level. They can farm insects without any processing of the products in the loop: feedstock for the insects is the by-products of their agriculture, aquaculture, and livestock production; reintroducing live larvae as a cheap feedstock for their fish or chicken production; and then using insect frass as soil fertilizer. Insects in general need a warm environment for optimal development, and the Asian climate provides this—an ideal range of temperature and humidity is available all year round in most locations under tropical conditions, representing a significant climate advantage for insect rearing, avoiding extremely expensive HVAC capital systems needed in colder climates. In small production units, optimal rearing conditions control (particularly temperature and humidity) can then be simplified to good ventilation for evacuating heat, humidity, and ammonia.

For large-scale farms producing a few thousand tons of insect ingredients per year, we can forecast different scenarios of development from business as usual to more ambitious and highly automated plants. Such scenarios will optimize the production costs and accelerate the recognition of the quality of insect-based products in terms of functionalities on health and performance (Shah et al., 2022). A thriving ecosystem of private and institutional investment interests exists in the Asia region to support growth.

## Funding and Investment

When comparing by region, the level of investments in the insect sector in Asia (a few tens of millions of USD) is low compared to Europe or North America, where projects of over hundreds of millions USD are underway.

According to J.Y. Chow, Singapore managing director of CREADEV, a globally operating investment company and one of Innovafeed investors, western projects are more mature: their inputs for substrates are well secured and their outputs with end use as well. Locking upstream and downstream flows constitutes major assets for the confidence of the financial markets in occidental projects, and, conversely, the lack of input and output security guarantee leads to some current reluctance of investors for Asian projects.

In J.Y. Chow’s view, insect as feed projects are now moving from innovation risk level to execution level, also known as the scale-up phase en route to market, while “patient and understanding” capital is still needed for this critical transition. He considers it does not give justice to insect protein to designate it as an *alternative protein* meal. He would rather call them *complementary protein*, and those who have been long enough in the feed industry understand well the need for optionality when the protein gap risk—especially in Asia—is an understatement. The insect industry understands very well the need for additional protein options, which is at the very heart of the development of the insect sector. As indicated above, protein per se will only represent one of the key aspects to increasingly consider insects as relevant ingredients for health, nutrition, and environmental sustainability. Increasing understanding will enable formulators to consider insect ingredients for their whole range of benefits.

The powerful opportunity presented by insect agriculture goes beyond the ecosystem services it can provide, and in increasingly recognized (Dangles and Casas, 2019). Asia holds important experience, and potential to harness this power with the right framework of incentives from the market, industry, academia, and policymakers. Can the potential of the insect agriculture opportunity develop to the extent that many anticipate?

## Conclusion and Prospects

In conclusion, while the legislative framework is far from being harmonized across ASEAN, the markets do not consider sustainability as a key element yet, and finally, while investors are cautious to invest in industrial projects in the region, opportunities for the insect industry in Southeast Asia are there: the historical background, the favorable climatic factor, and the strategic position at the heart of a region whose growing importance in animal nutrition is thriving.

Ultimately, the viability of the insect industry for animal and aquaculture feed may depend on its capacity to demonstrate an acceptable cost and performance equilibrium. Performance shall be considered and demonstrated as a whole set of parameters from well established (nutrition, health) to increasingly important ones like environmental sustainability.

Today, the price remains a deterrent to the wider adoption of insect-based ingredients. Key factors to enable the maturation of the industry will be productivity improvement through advanced animal husbandry and zootechny, continued product cost reduction through industry learning curve improvements, reduction of feed costs by widening the scope of authorized feedstuff, and through efficiency improvement in production processes and end product extraction (Malla and Opeyemi, 2018).

In addition, if the enhanced functional benefits and nutraceutical values (Surendra et al., 2020) showing promise are further demonstrated, different economic justifications are possible. At all costs, feed safety and public health shall remain important elements along the insect value chain, with good practices already implemented worldwide requiring continuous attention to mitigate related risks.

All these improvements will be able to occur with the right landscape of enabling partners, from academia to regulatory bodies, from policymakers to investors. International organizations like FAO and industry associations like AFFIA and the Global Roundtable for Insect Agriculture (GRIA) can play key convening roles in encouraging open information access for rapid and inclusive industry development.

## About the Authors

**Anne Deguerry** holds the position of Communication Director of Entofood, a pioneer BSF company based in Malaysia, since 2012. She served as a member of the AFFIA board from 2015 to 2020. A long-time advocate for sustainability involved in women’s community development, she firmly believes in cooperation between companies in the sector to promote the development of the insects as feed sector as a whole. She holds a master’s degree in commercial law and a postgraduate degree in European law from the University Lyon III in France.

**Nathan Preteseille** is an independent consultant working between Southeast Asia and Europe for the past 8 years and is specifically engaged with insects as food and feed from commercial, regulatory, and technical perspectives, in addition to different projects from farm to fork. He holds a master’s degree in biochemistry from INSA Toulouse in France, with a specialization in the food industry at ENSAT. His interest in insects is driven by the role they increasingly take in the provision of ecosystem services, especially in the food chain.

**Attawit Kovitvadhi** is a permanent lecturer at the Department of Physiology, Faculty of Veterinary Medicine, Kasetsart University in Bangkok, Thailand. He has also held positions as head of a small animal nutrition clinic, assistant dean of research, and acting director of the research support center at the Faculty of Veterinary Medicine in Bangkok, Thailand. He obtained his Doctor of Veterinary Medicine from Kasetsart University and his PhD in Animal Science and Technology from the University of Turin, Italy. His research interests include insect farming, using insects as feed, and extracting the high-value products from insects, specifically crickets and black soldier flies. Additionally, he also researches on the nutrition of poultry, dogs, cats, and exotic pets.

**David John Allan (adjunct) OAM** is the Founder and Executive Director of Spectrum—Sustainable Development Knowledge Network in Myanmar. His educational qualifications include a B.E.Chem (Hons), University of Queensland (UQ), an M.Eng.Sci., Monash University, and a Grad.Dip.Mgt., Deakin University. Since 2005, he has worked on sustainability strategy with the Myanmar people, with government, private sector, and communities towards common goals of more collective sustainable development, accessible energy, and environmental management. Since 2015, his focus has included applied research, public communications, and media on insects for food and feed in Myanmar, and various roles with AFFIA. Since 2018, he has been conferred an Associate Professor (adjunct) at the UQ Energy and Poverty Research Group. In 2022, he was awarded an Order of Australia Medal (OAM) for service to the international community of Myanmar.

**Sonevilay Nampanya** (FAO-RAP Livestock Development Officer): he has more than 15 years of experience working in animal health and production, livestock policy, and transboundary animal disease (TAD) control. In his capacity, he is leading and providing technical support to design and implement FAO-RAP livestock portfolio projects across the region from Afghanistan, Bangladesh, Mongolia, and Pakistan to the Pacific, contributing to support national and regional food security and poverty reduction. Sonevilay completed a degree in Animal Veterinary Bioscience with honors and a PhD from the University of Sydney, Australia.

**Scott Newman** (FAO-RAP Senior Animal Health and Production Officer) is a wildlife veterinarian and biologist with extensive international experience managing and implementing multidisciplinary One Health programs at the human–wildlife–livestock-ecosystem interfaces in Asia, Africa, North America, and Europe. He has worked in academic settings and for NGOs as a research scientist and has been with FAO for nearly 15 years focused on animal and zoonotic disease prevention and control, sustainable agricultural development and pastoralism, antimicrobial resistance and food safety, as well as wildlife ecology and natural resource management. He holds a PhD in Integrative Pathology, a doctorate in veterinary medicine (DVM), and a BS degree in Biology.
